# Greater early migration of a short-stem total hip arthroplasty is not associated with an increased risk of osseointegration failure: 5th-year results from a prospective RSA study with 39 patients, a follow-up study

**DOI:** 10.1080/17453674.2020.1732749

**Published:** 2020-02-28

**Authors:** Thilo Floerkemeier, Stefan Budde, Gabriela v. Lewinski, Henning Windhagen, Christof HurSchler, Michael Schwarze

**Affiliations:** a Department of Orthopaedic Surgery, Hannover Medical School, Germany;;; b Laboratory for Biomechanics and Biomaterials, Hannover Medical School

## Abstract

Background and purpose — Short-stem hip arthroplasty has been a viable alternative to standard stems for the treatment of hip osteoarthritis for over 10 years. This study assessed whether a correlation existed between a greater initial increase in implant migration and inferior clinical outcomes at 5 years postoperatively. Results on these patients after 2 years have been published previously.

Patients and methods — Radiostereometry and clinical scoring were undertaken after surgery and at 3, 6, 12, and 24 months, and 5 years postoperatively. The migration and the clinical outcomes data from the patients with initial migrations at 3 months above the 75th percentile (≥ 75% group) were compared with those with migrations at 3 months of less than the 75th percentile (< 75% group).

Results — Between 3 months and 5 years after surgery, the mean resultant implant migrations were 0.40 mm (SD 0.32) in the ≥ 75% group and 0.39 mm (SD 0.25) in the < 75% group. The mean Harris Hip Scores and SF-36 physical scores at 5 years postoperatively were 100 (SD 0.4) and 44 (SD 12), respectively, for the ≥ 75% group and 99 (SD 2) and 50 (SD 10), respectively, for the < 75% group. The differences between the patient groups were not statistically significant.

Interpretation — There was no correlation between a greater initial migration and inferior clinical outcomes at 5 years postoperatively. Despite a greater initial migration, there were no risks of early aseptic loosening and inferior midterm clinical outcomes associated with a short-stem implant with a primary metaphyseal anchorage.

Short-stem hip prostheses are commonly used to treat hip osteoarthritis, especially among younger patients (Thorey et al. 2013). The proposed advantages of using these prostheses include more physiological proximal load transfers to the surrounding bone that potentially reduce stress shielding and provide better options should revision surgery become necessary (Floerkemeier et al. 2017). Furthermore, implanting a short-stem prosthesis with a partial resection of the femoral neck is particularly compatible with minimally invasive surgery, which not only minimizes the length of the skin incision, but, more importantly, reduces muscle and tendon damage (Mjaaland et al. 2015). Findings from studies involving radiostereometric analysis (RSA) of short stems with primary metaphyseal fixations have suggested greater migration during the first postoperative months, followed by stabilization (Budde et al. 2016, Schwarze et al. 2018). Data describing the migration characteristics of this specific short-stem implant beyond 2 years of follow-up have not been reported (Schwarze et al. 2018). This previous study focused on the effect of surgical approach and did not find an influence on migration or clinical results. Furthermore, it remains questionable whether a greater initial migration of short-stem implants during the first postoperative weeks represents a risk factor for later loosening. In addition, it is unclear whether a greater initial migration is associated with inferior long-term functional outcomes.

Hence, this study assessed whether a greater initial migration at the first follow-up assessment after a short-stem total hip arthroplasty (a) signifies a further increase in migration at the midterm follow-up assessment, which would indicate a risk of later aseptic loosening, and (b) is associated with inferior subsequent clinical outcomes and pain.

## Patients and methods

The previous study described 2-year follow-up data from 60 patients who underwent total hip arthroplasty using a Metha short-stem prosthesis (Schwarze et al. 2018). To obtain 5-year follow-up data, the same patient cohort was followed for an additional 3 years. The initial 2-year randomized controlled study enrolled 60 patients (34 women and 26 men) with a mean age of 59 years (36–72) and a mean BMI of 26 (21–37) between February 2010 and June 2013. These patients provided written informed consent prior to the operation and before they participated in the 5-year follow-up study. The study’s original inclusion criteria were patients aged 30–75 years with progressive osteoarthritis of the hip that was confirmed by analyzing plain radiographs. The study’s exclusion criteria were neurological disorders, cardiovascular disorders affecting ambulation, sensorimotor disorders, previous bone surgery on the affected joint, allergic reactions to the implant materials, revision surgery, and an unwillingness to participate in the extension of the study.

The present study analyzed the use of the Metha short-stem implant (Aesculap AG, Tuttlingen, Germany), which is representative of the partial neck-sparing group of short-stem implants (Lombardi et al. 2009). Likewise, Feyen and Shimmin (2014) classified this implant as a short-stem implant with a subcapital osteotomy. All of the patients received appropriately sized (sizes 1–7) cementless short-stem hip implants. The stem was made of a titanium forged alloy (Ti4Al6V) coated with pure titanium and a 20-µm layer of calcium phosphate in the proximal region, and had a polished tip. The patients included in this study received the nonmodular version of the implant that had a caput-collum-diaphyseal angle of 120°, 130°, or 135°. A Plasmacup SC press-fit acetabular component (Aesculap AG, Tuttlingen, Germany) was used with either a polyethylene or a ceramic insert; ceramic heads of 32-mm diameter were used exclusively.

Surgery was performed by 1 of 5 experienced surgeons using either a conventional transgluteal lateral Hardinge approach or an anterolateral modified Watson-Jones approach. The anesthesia protocol, insertion of the tantalum beads that provided a reference for the migrations calculated using RSA, postoperative pain management protocol, rehabilitation program, and the discharge criteria have been described previously (Schwarze et al. 2018). The precision of the RSA setup was determined through duplicate examinations of 15 patients (Table 1, see Supplementary data).

The 5-year follow-up appraisals between February 2015 and June 2018 involved examinations of the RSA images and clinical investigations that comprised assessments using the Harris Hip Score (HHS), the physical functioning scale of the Short Form-36 (SF-36), the mental health domain of the SF-36, perceived pain measured in a visual analog scale (VAS), and radiological examinations. A score of 0 on the VAS pain scale corresponds to “no pain,” while 10 corresponds to “maximum pain imaginable.” Implant migration was calculated relative to the first postoperative images that were captured at 3–10 days postoperatively. The implant surface models required for model-based RSA were obtained using reverse engineering techniques. The calibration box’s coordinate system served as the reference for the migration measurements, where x was positive in the medial direction, y was positive in the cranial/proximal direction, and z was positive in the anterior direction. In addition to migration along these axes, implant migration relative to the surrounding bone was calculated as the magnitude of the resultant movement vector (which is (Tx²+Ty²+Tz²)^0.5^) of the implant’s geometric center, and it was, therefore, always positive. The RSA parameters and procedures (Table 1, see Supplementary data) were defined in accordance with standard guidelines (ISO 2013).

As relatively large initial migrations of implants were observed at 3 months postoperatively in the initial study, which had a 24-month follow-up duration, the patient population was divided into 2 groups for further analysis. One group comprised patients with initial resultant migrations below the 75th percentile, that is, < 1.43 mm, (the < 75% group), and the other group comprised patients with initial resultant migrations that were equal to or above the 75th percentile, that is, ≥ 1.43 mm (the ≥ 75% group). These groups were compared to investigate relationships between the extent of the initial resultant migration and the midterm clinical outcomes.

### Statistics

The clinical scores in the groups with low (< the 75th percentile) and high (≥ the 75th percentile) initial migration were compared at each follow-up using two-sided Student’s t-tests with significance levels of α = 0.05. To compare clinical scores and resultant implant migration between follow-up intervals within each group, we used paired t-tests that used a Bonferroni-corrected initial significance level of 0.05.

### Ethics, registration, funding, and potential conflicts of interest

The local ethics committee approved this study (Amendment to Institutional Review Board No. 4565, February 2015). The initial 2-year randomized controlled study was registered in the German Clinical Trials Register (DRKS00010421). The study was funded by Aesculap AG Tuttingen, Germany. The authors TF, GvL, and HW are paid instructors in lecture courses for Aesculap AG.

## Results

**Figure 1.**
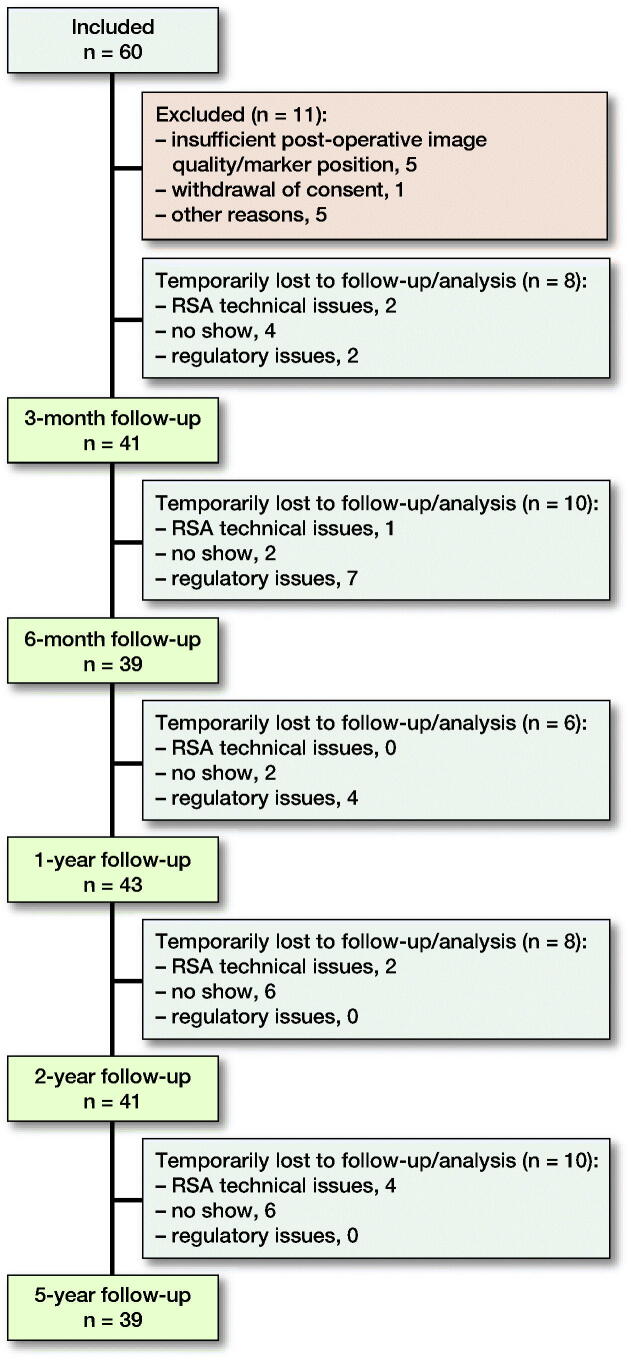
Flowchart of the 60 study participants and the number of patients at each follow-up assessment. The technical issues associated with the radiostereometric analysis included marker occlusion, caused by unusual positioning of ovarian/testicular radiation protection, and insufficient image quality.

Of the 60 patients initially recruited, 11 were excluded from the analysis at the 24-month follow-up stage for a variety of reasons (Schwarze et al. 2018). A further 10 of the 49 remaining patients were lost to follow-up at the 5-year follow-up stage (Figure 1). 252 RSA image pairs were analyzed successfully.

### Implant migration

The 2 criteria that defined the quality of the RSA images, namely, the rigid-body error (median: 0.140 mm [0.017–0.316]) and the condition number (median: 24 [13–118]), were within acceptable ranges (< 0.35 mm and < 120, respectively). At 60 months postoperatively, the mean (standard deviation [SD]) resultant migration was 1.12 mm (SD 1.21; 0.15–5.05), which did not statistically significantly differ from the migrations determined at the earlier follow-up intervals, namely, 3, 6, 12, and 24 months (Figure 2). The mean initial resultant migrations were 2.71 mm (SD 1.56) in the ≥ 75% group and 0.47 mm (SD 0.30) in the < 75% group (Figure 2). At 60 months postoperatively, the resultant migrations were 3.08 mm (SD 1.41) in the ≥ 75% group and 0.61 mm (SD 0.33) in the < 75% group. With the 3-month follow-up used as baseline, the resultant migrations were 0.41 mm (SD 0.32) in the ≥ 75% group and 0.39 mm (SD 0.25) in the < 75% group after 60 months. The results did not statistically significantly differ between groups at any follow-up.

Figure 2.Box-plots showing resultant implant migration (A) in all patients during follow-up, (B) during follow-up, categorized according to the initial migration percentile, (C) in all patients in relation to the first postoperative follow-up, and (D) in relation to the 3-month follow-up. ν represents the ≥ 75% group, and ν represents the < 75% group.

**Figure:**
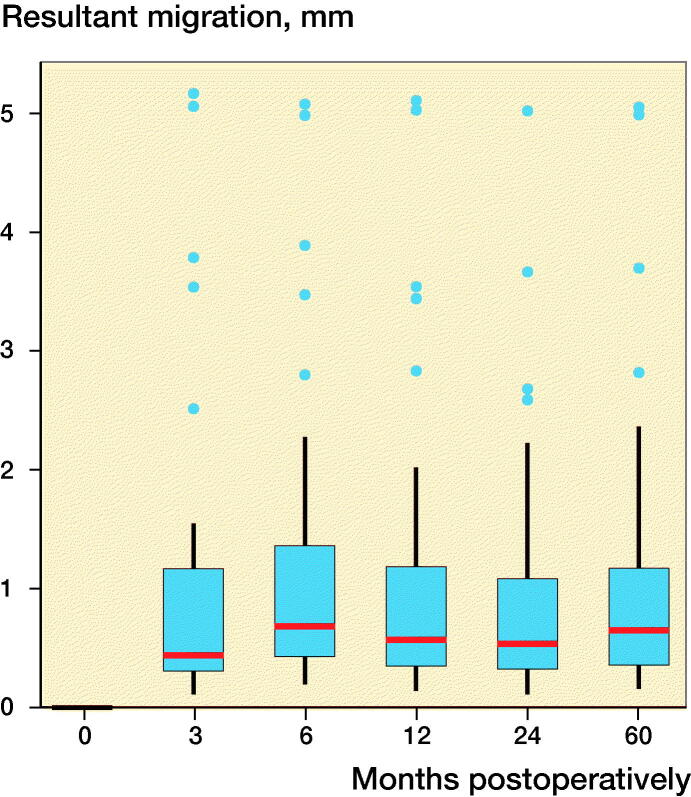

**Figure:**
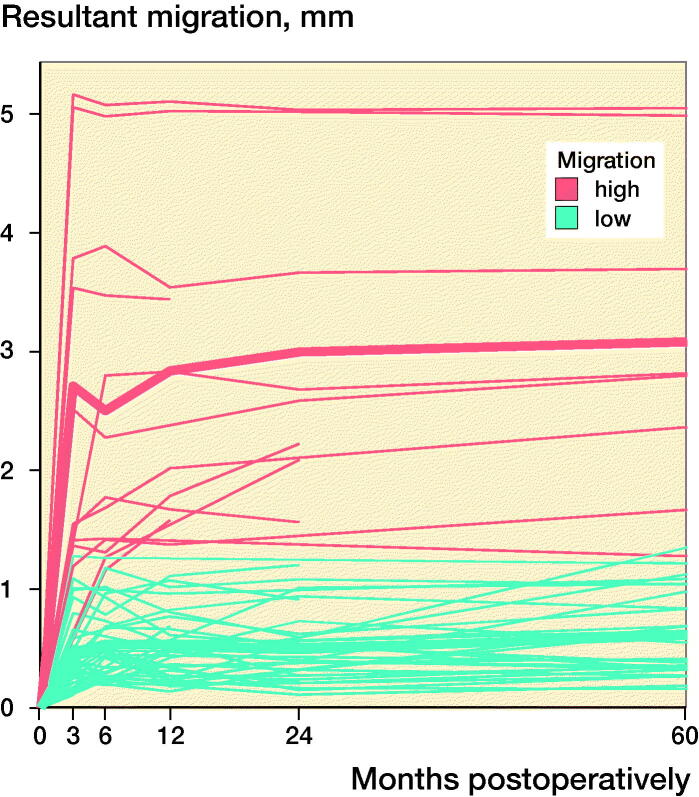

**Figure:**
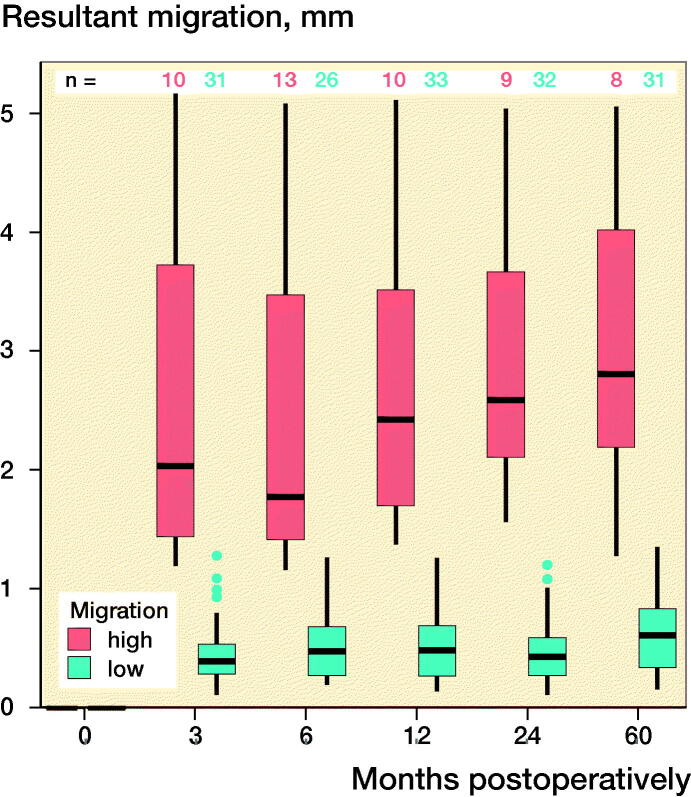

**Figure:**
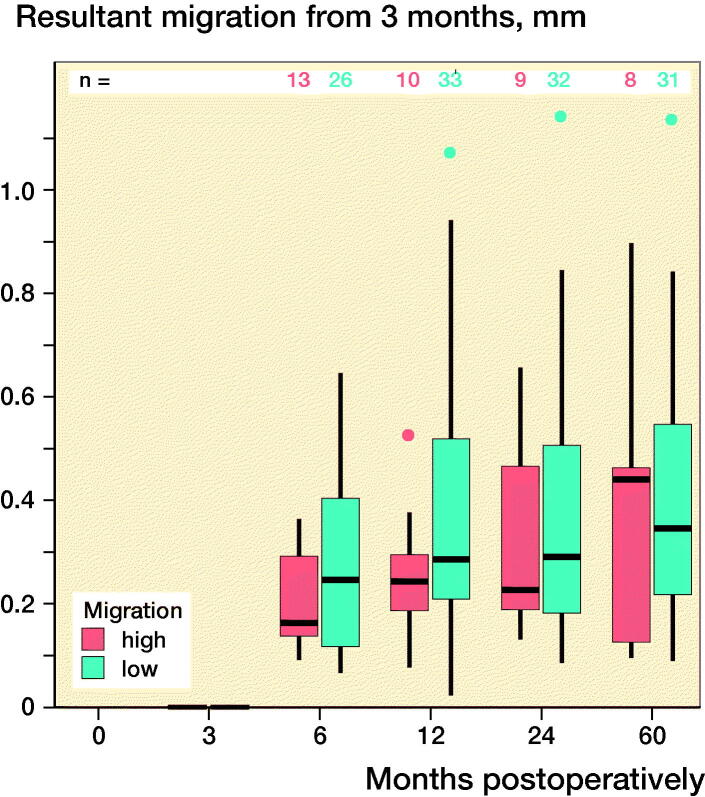

The major contribution to the resultant migration is subsidence in the distal direction, followed by a lateral translation for the ≥ 75% group and a posterior translation for the < 75% group. The largest rotations were observed about the proximal–distal axis (Table 2, see Supplementary data).

### Clinical outcomes

Both groups’ clinical scores increased postoperatively and plateaued at 12 months postoperatively (Figure 3). At 5 years postoperatively, the mean (SD) HHSs for the ≥ 75% and < 75% groups were 100 (0.4) and 99 (2), respectively, and the mean (SD) VAS scores for the ≥ 75% and < 75% groups were 0.8 (1.0) and 1.3 (1.6), respectively. The mean (SD) physical functioning scale of the SF-36 and the mental health domain of the SF-36 were 44 (12) and 57 (2) for the ≥ 75% group and 50 (10) and 51 (10) for the < 75% group at 5 years postoperatively. Overall, there were no statistically significant differences between the groups in relation to the mean HHSs and the SF-36 and VAS scores, and the extent of the initial migration had no significant effects on the clinical scores (Figure 3).

Figure 3.Clinical scores at each follow-up interval. There were no significant differences between the groups. ν represents the ≥ 75% group and ν represents the < 75% group. (Harris Hip score; SF-36-P: Physical functioning scale of the Short Form-36; SF-36-M: Mental health domain of the Short Form-36; VAS: Visual analog scale pain score).

**Figure:**
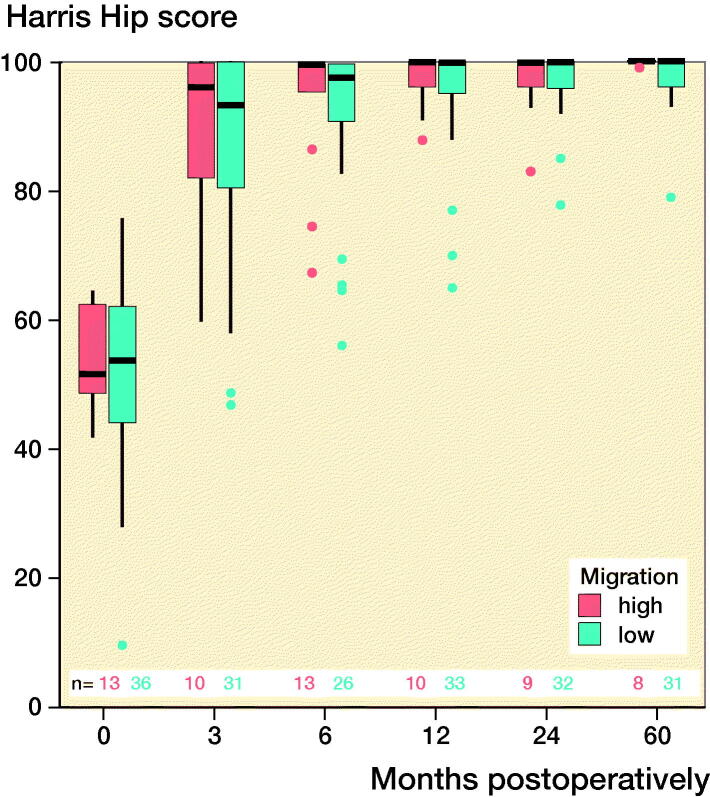

**Figure:**
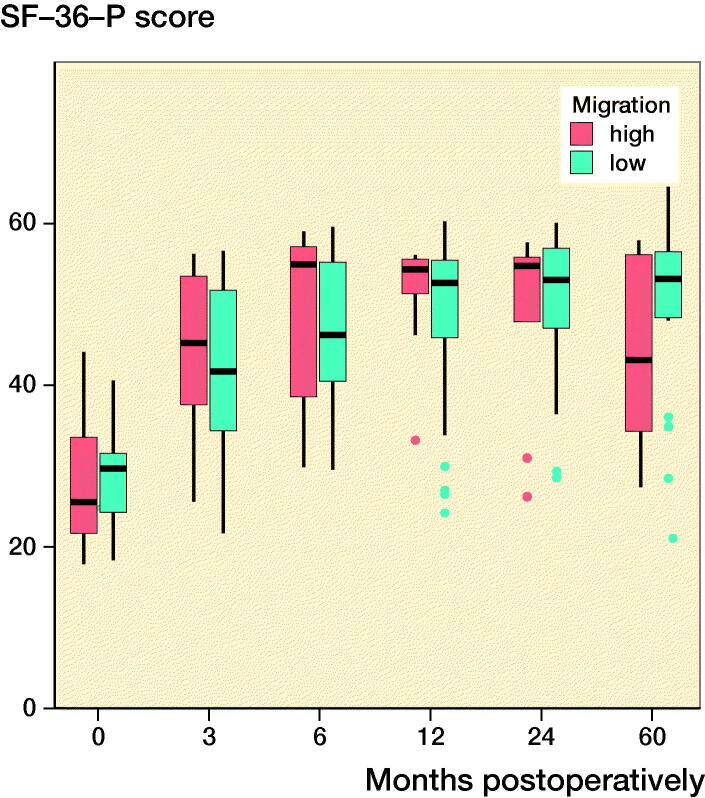

**Figure:**
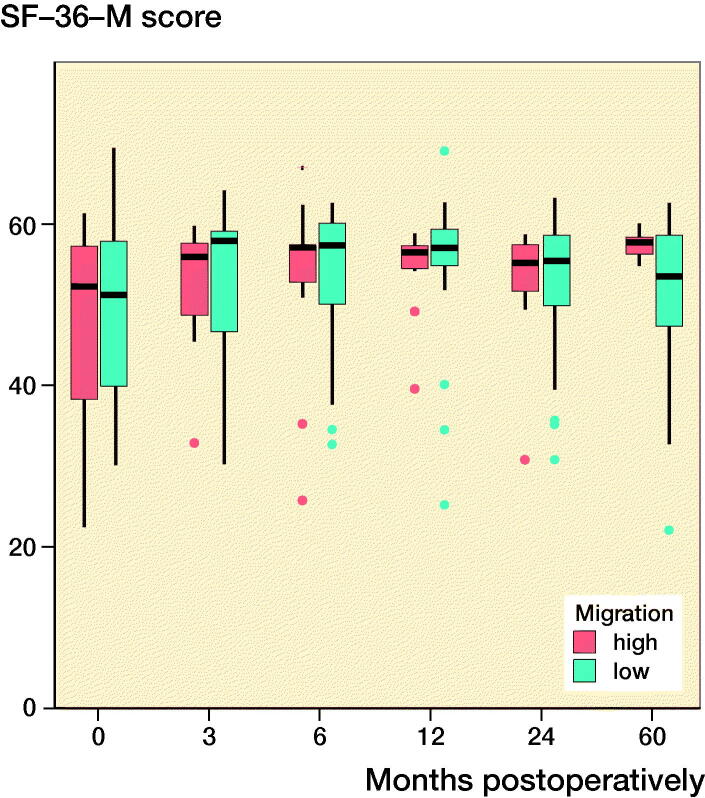

**Figure:**
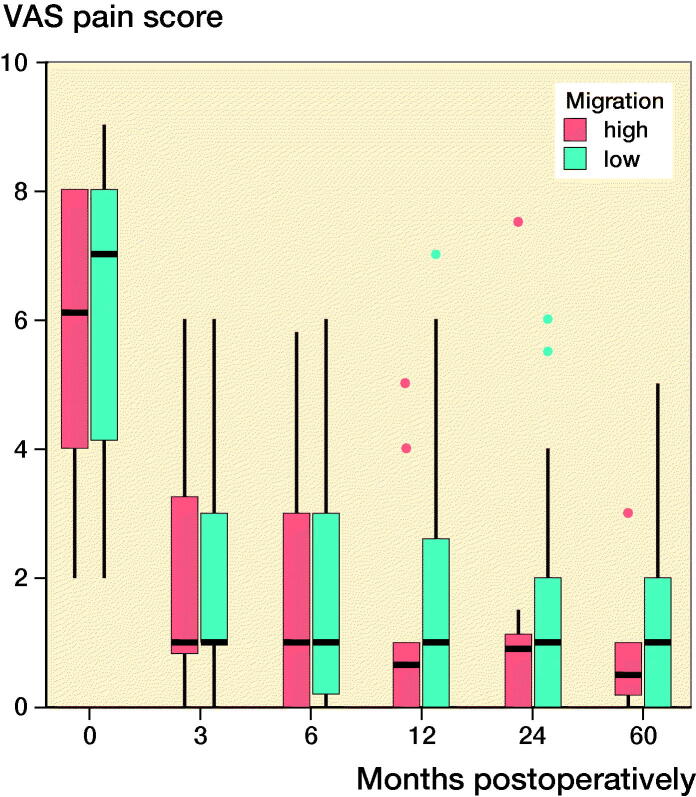

## Discussion

The RSA findings were similar between the < 75% group and the ≥ 75% group with respect to additional migration; therefore, a greater initial migration was not associated with steadily increasing migration and an increased risk of aseptic loosening. Furthermore, our data did not reveal an association between a greater initial migration and inferior midterm clinical outcomes. We observed that the prosthesis was stable after the first 3 months and that migration did not increase significantly over the 5-year follow-up period. Thus, we did not find that a greater initial migration led to a higher risk of early aseptic loosening or insufficient osseointegration. In fact, good secondary osseointegration and excellent clinical outcomes were observed, regardless of the magnitude of the initial migration.

Several publications from studies that used highly accurate RSA have described the migration of uncemented total hip implants (Table 3, see Supplementary data). When comparing studies in relation to the migration patterns associated with different stem systems, it must be remembered that there are several ways to express implant migration data using, for example, the maximum total point motion, orthogonal translations and rotations, or the resultant migration. Subsidence, which is defined as distal translation along the y-axis, was the major contributor to the resultant migration observed among the patients in our study, and it was used to undertake more robust comparisons with the data from previously published studies (Table 3, see Supplementary data). Compared with other uncemented stem systems, the Metha short-stem system seems to possess less primary stability. The key conclusion that can be drawn is that the Metha short-stem implant with a primary metaphyseal anchorage provides stability and enables osseointegration after an initial phase of about 3 months. Even among patients with greater initial migrations of ≥ 1–2 mm immediately after the operation, secondary osseointegration is possible.

Kärrholm et al. (1994) established a method for predicting long-term aseptic loosening that was based on the migration of hip stems that exceeded 1.2 mm after 2 years. This method was established using cemented long-stem implants that have different underlying fixation principles compared with uncemented short-stem devices, because the bone-loading patterns differ and initial settling of the implant into the compacted bone can be expected (Salemyr et al. 2015). Our study’s results suggest that a value of 1.2 mm after 2 years is not directly applicable for short stems. These findings confirm the conclusion given by Kroell et al. (2009) that the initial migration patterns associated with short-stem implants and the subsequent secondary stabilization may not predict long-term survival. Similar to our findings, these authors found that excellent secondary osseointegration may occur even among patients with initial migrations of > 2.0 mm within the first 3 months.

In our first study, the stem survival rate was 97% after 2 years (n = 2 revisions due to infection). After 5 years, no further revisions were necessary. The cases in the ≥ 75% group did not show any clinical signs of loosening at 5 years’ follow-up. The stems of these patients have been proven to be stable without clinical or radiological hint of further subsidence or loosening.

Good clinical outcomes were evident at 60 months regarding both the HHS and SF-36 scores. Whilst other publications found a constant development of the SF-36 physical score towards the 5-year follow-up (Nebergall et al. 2016), the ≥ 75% group revealed a drop at 5 years in that score compared with 2 years. However, due to the small sample size in the group, this should not be overrated, since the difference for the < 75% group is not statistically significant. Regarding the specific stem of our study, several authors have published data describing midterm clinical outcomes at similar follow-up intervals after implanting the Metha short-stem device: Thorey et al. (2013) showed that after a mean follow-up duration of 5.8 years, the mean (SD) HHS had increased from 46 (17) preoperatively to 90 (5) postoperatively, the mean Hip dysfunction and Osteoarthritis Outcome Score (HOOS) had improved from 55 (16) preoperatively to 89 (10) postoperatively, and the Kaplan–Meier survival rate was about 98%. Lacko et al. (2014) showed that within a group that received Metha short-stem implants, the mean preoperative and postoperative HHSs were 42 (10) and 94 (5), respectively, and subsidence of the stem had occurred in 1 patient. Wittenberg et al. (2013) presented clinical and radiological data from 250 patients who received Metha stem devices, and they showed that with a mean follow-up duration of 4.9 years, the average HHS was 97 points, the 5-year Kaplan–Meier survival rate was 96.7%, and 85% of the patients were very satisfied, 14% were satisfied, and 1% were dissatisfied with the treatment.

Our study has several limitations. First, the postoperative RSA image set was captured after initial weight-bearing. Thus, it is possible that initial settling of the implant had occurred previously, which was not considered in the data presented. Unfortunately, organizational factors in the clinic meant that no other procedure was possible. Second, a high number of the patients dropped out of the study during follow-up. On average only two-thirds of our patient population were examined at each follow-up. Nonetheless, compared with other RSA studies, the number of patients is quite high and in accordance with requirements (ISO 2013) (Table 3, see Supplementary data).

In summary, our data demonstrated that there were no statistically significant differences between the group of patients with minor initial implant migrations and the group of patients with greater initial implant migrations with respect to additional migration over 5 years. Therefore, a greater initial migration was not associated with an increased risk of aseptic loosening and subsequent insufficient osseointegration. Furthermore, this study’s findings did not demonstrate an association between a greater initial implant migration and inferior midterm clinical outcomes. Overall, the migration of the Metha short-stem implant generated promising data in this study, without hints of aseptic loosening.

## Supplementary Material

Supplemental Material
