# Supplementary material for: Greater early migration of a short-stem total hip arthroplasty is not associated with an increased risk of osseointegration failure: 5th-year results from a prospective RSA study with 39 patients, a follow-up study
Source: Acta Orthop. 2020 Feb 28;91(3):266–71. doi: 10.1080/17453674.2020.1732749 (PMC8023937; doi:10.1080/17453674.2020.1732749)
Supplement: Supplemental Material [file IORT_A_1732749_SM5532.pdf]

Supplementary data

Table 1. RSA parameters and equipment used for image acquisition and analysis according to ISO 16087–2013

Parameter	Value
Calibration cage	CarbonBoxLeiden10Hannover
X-ray tubes	2x SRO3310 ROT 360 (Philips)
Tube voltage/current	90 kV / 12.5 mAs
Angle between X-ray paths	40°
X-ray cassette	36x43 cm IP Cassette Type CC (Fuji)
Cassette digitizer	PCR Eleva Corado (Philips), resolution: 125dpi
Threshold	
Condition number	120
Mean rigid body error	0.35 mm
RSA software version	Medis Specials Model-based RSA 3.2/3.31
Precision (95% confidence interval) by double examination on 15 patients	
Translation, mm	
x	0.014 (–0.072 to 1.000)
y	–0.019 (–0.166 to 0.128)
z	–0.046 (–0.275 to 0.183)
Rotation, °	
x	0.03 (–0.74 to 0.80)
y	0.09 (–3.03 to 3.21)
z	–0.01 (–0.22 to 0.20)
Resultant migration, mm	0.126 (–0.038 to 0.290)

Table 2. Mean migration and standard deviation for each translation and rotation at each follow-up. Values are mean (SD). The x-axis is positive in the medial direction, the y-axis is positive in the cranial/proximal direction, and the z-axis is positive in the anterior direction

Follow-up, months	Translation (mm) x	Translation (mm) y	Translation (mm) z	Rotation (°) x	Rotation (°) y	Rotation (°) z	Resultant migration (mm)
< 75% group							
3	–0.03 (0.20)	–0.21 (0.34)	–0.04 (0.34)	0.39 (1.6)	2.3 (6.8)	–0.18 (0.54)	0.47 (0.30)
6	0.00 (0.23)	–0.18 (0.35)	–0.09 (0.42)	0.57 (1.9)	4.3 (8.4)	0.00 (0.64)	0.54 (0.31)
12	–0.05 (0.25)	–0.22 (0.34)	0.00 (0.39)	0.77 (2.0)	3.7 (8.4)	–0.17 (0.54)	0.54 (0.30)
24	0.03 (0.25)	–0.17 (0.30)	–0.18 (0.34)	0.93 (1.9)	5.1 (8.1)	–0.11 (0.62)	0.49 (0.28)
60	0.04 (0.30)	–0.24 (0.35)	–0.21 (0.42)	0.36 (1.9)	4.3 (7.8)	–0.16 (0.93)	0.61 (0.33)
≥ 75% group							
3	–0.55 (0.89)	–2.4 (1.6)	–0.18 (0.55)	–1.2 (1.0)	–2.5 (5.0)	0.28 (1.0)	2.7 (1.6)
6	–0.50 (0.85)	–2.2 (1.6)	–0.30 (0.55)	–1.3 (1.9)	–1.1 (5.7)	0.27 (1.2)	2.5 (1.4)
12	–0.83 (0.82)	–2.6 (1.4)	–0.05 (0.34)	–0.5 (2.3)	–2.4 (5.9)	0.40 (1.3)	2.8 (1.4)
24	–0.75 (0.91)	–2.7 (1.4)	0.09 (0.70)	–0.1 (2.9)	–2.0 (3.6)	0.23 (1.7)	3.0 (1.3)
60	–0.64 (1.01)	–2.6 (1.9)	–0.07 (0.56)	1.1 (3.5)	–0.4 (7.2)	0.84 (2.0)	3.1 (1.4)
All							
3	–0.16 (0.50)	–0.73 (1.3)	–0.07 (0.40)	0.02 (1.6)	1.1 (6.7)	–0.07 (0.70)	1.0 (1.2)
6	–0.17 (0.57)	–0.83 (1.3)	–0.16 (0.47)	–0.04 (2.1)	2.5 (8.0)	0.09 (0.86)	1.2 (1.3)
12	–0.23 (0.55)	–0.77 (1.2)	–0.01 (0.38)	0.46 (2.1)	2.3 (8.3)	–0.04 (0.80)	1.1 (1.2)
24	–0.14 (0.56)	–0.72 (1.2)	–0.12 (0.45)	0.70 (2.1)	3.5 (7.9)	–0.04 (0.94)	1.0 (1.2)
60	–0.10 (0.58)	–0.71 (1.3)	–0.18 (0.45)	0.51 (2.3)	3.4 (7.8)	0.05 (1.3)	1.1 (1.2)

Table 3. Non-comprehensive summary of studies involving radiostereometric analysis that analyzed the y-axis migration of different cementless total hip arthroplasty stems

Authors	Stem implant	Classification I [a]	II [b]	Follow-up, months 3	6	12	24	36	60	Total n
Nysted et al. (2014)	ABG-I, Stryker, USA	C	IV	–	–	–	–	–	–0.03	43
Matejcic et al. (2015)	Scyon THA, Scyon Orthopaedics, Switzerland	C	IIIb	–	–0.03	–0.07	–0.05	–	–0.04	15
Nebergall et al. (2016)	Taperloc, Biomet Inc., Warsaw, USA	C	IV	–	–0.11	–0.09	–0.07	–0.05	–0.03	41
Nysted et al. (2014)	Unique femoral stem, Stryker, USA	C	IIIb	–	–	–	–	–	–0.13	47
Röhrl et al. (2006)	CFP, Link, Germany	B	IIIa	–0.10		–0.06	–0.08	–	–	13
Lazarinis et al. (2013)	CFP, Link, Germany	B	IIIa	–0.10	–	–0.09	–0.13	–	–	26
Nieuwenhuijse et al. (2012)	CUT, ESKA, Germany	A	II	–0.10	–0.09	–0.10	–0.13	–0.08	–0.09	39
Mahmoud et al. (2017)	Proxima, DePuy, USA	B	III	–0.22		–0.23	–0.22	–	–	28
Budde et al. (2016)	Nanos, Smith & Nephew, UK	B	IIIa	–0.23	–0.20	–0.17	–0.22	–	–	14
Weber et al. (2014)	Furlong HAC stem, JRI Ltd., UK	C	IV	–0.31	–	–0.33	–0.26	–	–0.29	25
Ferguson et al. (2018)	MiniHip (Head), Corin Group, Great Britain	C	III	–	–0.03	–0.16	–0.26	–	–	20
Flatøy et al. (2016)	Taperloc, HA-coated, Biomet Inc., Warsaw, USA	C	IV	–0.30	–	–	–	–	–	15
Klein et al. (2019)	CFP, Link, Germany	B	IIIa	–	–	–	–0.30	–	–	39
McCalden et al. (2015)	Synergy, Smith & Nephew, UK	C	V	–	–	–	–0.32	–	–	20
Hjorth et al. (2016)	Bi-Metric, Compaction, Biomet Inc., Warsaw, USA	C	IV	–0.96	–	–0.82	–0.32	–	–0.67	18
Flatøy et al. (2016)	Taperloc, BM-coated, Biomet Inc., Warsaw, USA	C	IV	–0.33	–	–	–	–	–	18
Sesselmann et al. (2018)	Cerafit, Ceraver, France	C	IV	–	–0.36	–0.33	–0.33	–	–	26
Acklin et al. (2016)	Fitmore, Zimmer, Switzerland	C	IIIb	–0.39	–0.36	–0.40	–0.39	–	–	24
Simpson et al. (2010)	Furlong HAC stem, JRI Ltd., UK	C	IV	–	–0.27	–0.30	–0.40	–	–	23
Hoornenborg et al. (2018)	SL-PLUS HA-coated, Smith&Nephew, UK	C	IV	–0.53	–0.48	–0.50	–0.46	–	–	21
Klein et al. (2019)	Corail, Depuy, USA	C	IV	–	–	–	–0.50	–	–	38
Hjorth et al. (2016)	Bi-Metric, Broaching, Biomet Inc., Warsaw, USA	C	IV	–1.05	–	–0.99	–0.54	–	–0.88	18
Campbell et al. (2011)	Corail, Depuy, USA	C	IV	–	–0.73	–0.62	–0.58	–	–	20
Ferguson et al. (2018)	MetaFix (Head), Corin Group, Great Britain	C	IV		–0.29	–0.45	–0.62			18
**This study**	**Metha, Aesculap, Germany**	**B**	**IIIa**	**–0.73**	**–0.83**	**–0.77**	**–0.72**	**–**	**–0.71**	**39**
Aro et al. (2018)	ABG-II, Stryker, USA	C	IV	–	–	–	–0.73	–	–	53
Hoornenborg et al. (2018)	SL-PLUS non-coated, Smith&Nephew, UK	C	IV	–0.68	–0.73	–0.78	–0.73	–	–	16
Rutherford et al. (2019)	Corail with graft (DePuy Synthes, USA)	C	IV	–	–	–	–0.76	–	–	31
Rutherford et al. (2019)	Corail without graft (DePuy Synthes, USA)	C	IV	–	–	–	–0.93	–	–	41
McCalden et al. (2015)	SMF, Smith & Nephew, UK	B	IIIa	–	–	–	–0.94	–	–	18
Weber et al. (2014)	Furlong Active stem JRI Ltd. UK	C	IV	–0.99	–	–0.98	–0.98	–	–1.04	25
Simpson et al. (2010)	Furlong Active stem JRI Ltd. UK	C	IV	–	–0.99	–1.07	–1.11	–	–	20
Edmondson et al. (2014)	K2, Global Orthopaedic Technology, Australia	C	IIIa	–1.28	–	–1.91	–	–	–	130
Edmondson et al. (2014)	Apex, Global Orthopaedic Technology, Australia	C	IV	–2.12	–	–2.50	–	–	–	130

[a] The implants are categorized as (A) collum stems, (B) partial collum-preserving stems, and (C) collum-resecting stems, according to Jerosch (2012).

[b] The implants are categorized as (II) mid-head resection, (III) short stems with (a) subcapital osteotomy or with (b) ‘standard’ osteotomy, (IV) standard stem, and (V) diaphyseal fixation according to Feyen and Shimmin (2014).

Migrations at each reported follow-up assessment are presented. For better comparability, only subsidence data are presented in ascending order at the follow-up durations that were closest to 24 months. The number of patients (n [total]) refers to the minimum number of patients reported at any follow-up interval up to 2 years.
